# Cardiovascular Disease Outcomes Associated with Obstructive Sleep Apnea in Diabetics: A Systematic Review and Meta-Analysis

**DOI:** 10.3390/diseases11030103

**Published:** 2023-08-07

**Authors:** Ganesh Bushi, Bijaya Kumar Padhi, Muhammed Shabil, Prakasini Satapathy, Sarvesh Rustagi, Keerti Bhusan Pradhan, Zahraa Haleem Al-qaim, Jagdish Khubchandani, Ranjit Sah, Sanjit Sah, Ayush Anand

**Affiliations:** 1 Global Center for Evidence Synthesis, Chandigarh 160036, India; ganyganesh313@gmail.com (G.B.); bkpadhi@gmail.com (B.K.P.); mohdshabil99@gmail.com (M.S.); contact@gces.network (P.S.); 2 Department of Community Medicine and School of Public Health, Postgraduate Institute of Medical Education and Research, Chandigarh 160012, India; 3 School of Applied and Life Sciences, Uttaranchal University, Dehradun 248007, India; sarveshrustagi@uumail.in; 4 Department of Healthcare Management, Chitkara Business School, Chitkara University Punjab, Patiala 140401, India; keerti@chitkara.edu.in; 5 Department of Anesthesia Techniques, Al-Mustaqbal University College, Hillah 51001, Iraq; zahraahaleem@uomus.edu.iq; 6 Department of Public Health Sciences, New Mexico State University, Las Cruces, NM 88003, USA; 7 Department of General Practice and Emergency Medicine, Tribhuvan University Teaching Hospital, Kathmandu 46000, Nepal; ranjitsah@iom.edu.np; 8 Global Consortium for Public Health and Research, Datta Meghe Institute of Higher Education and Research, Jawaharlal Nehru Medical College, Wardha 442001, India; sanjitsah101@gmail.com; 9 Department of Medicine, B.P Koirala Institute of Health Sciences, Dharan 56700, Nepal; ayushanandjha@gmail.com

**Keywords:** sleep apnea, cardiovascular diseases, diabetes, stroke, comorbidity

## Abstract

Background: There is significant pathogenic and epidemiological overlap between diabetes and obstructive sleep apnea (OSA). This systematic review aimed to ascertain the association between OSA and cardiovascular disease (CVD) in a diabetic population. Methods: The study protocol was registered with PROSPERO (CRD42023404126). On 15 July 2023, a comprehensive search of the literature was performed in PubMed, EBSCO, Scopus, ProQuest, and Web of Science, using keywords and synonyms of OSA, diabetes, and CVD, coupled with specific terms for different CVDs. Only observational studies that reported CVD events in diabetics (with and without OSA) were included. The quality of the studies included in the analysis was assessed using the Newcastle–Ottawa Scale. Results: In the primary literature search, 8795 studies were identified, of which 9 met the inclusion criteria and included 17,796 participants. Eight studies were eligible for meta-analysis, and a pooled risk ratio (RR) of 1.29 (95% CI = 0.91–1.83) was found for developing CVD in diabetics with OSA at a 95% prediction interval of 0.30–5.60. The included studies showed significant heterogeneity with an I^2^ value of 91%. Conclusion: These findings show the possible association between OSA and diabetes and their impact on CVDs. Identifying and managing OSA in individuals with diabetes at an early phase could potentially reduce the risk of CVDs and its related complications.

## 1. Introduction

The global burden of diabetes is increasing, affecting nearly 500 million people in 2017 [1]. By 2025, diabetes is expected to contribute to the death of 1.59 million people globally [1]. One of the major contributors to diabetes-related mortality is CVDs affecting approximately one-third of people with diabetes [2,3]. Though the mortality due to CVDs has decreased over time due to effective secondary prevention strategies, the increasing prevalence of diabetes continues to contribute to the rising burden of CVDs [1,2,4]. OSA is a chronic condition that is increasingly becoming more prevalent. This rise in cases could be attributed to factors such as its potential connection to metabolic syndrome or an increased awareness of the OSA among people, leading to more diagnoses [5,6]. More than 900 million adults have been affected by OSA globally, with about two-fifths in the moderate to severe category [7]. The global prevalence ranges from 9% to 38%, with males (13% to 33%) more likely to be affected than females (6% to 19%) [5]. The elderly and obese individuals are also at higher risk. These latest estimates are higher than those reported in studies from the early 21st century and reflect an increasing prevalence globally [8]. The increased prevalence of OSA is of concern as the risk of obesity, dyslipidemia, diabetes, insomnia, and excessive daytime sleepiness is higher among individuals with OSA [9,10]. Also, OSA can adversely affect the hypoxia–reoxygenation system and sleep cycle, increasing several markers of inflammation, oxidative stress, endothelial dysfunction, and sympathetic activity, thereby increasing the risk of adverse cardiovascular events [11]. Numerous clinical studies have reported elevated levels of inflammatory biomarkers in individuals with OSA. Additionally, researchers have developed animal models of OSA and assessed the changes in biomarkers. Several of these experiments have provided evidence that intermittent hypoxia can activate inflammatory pathways, potentially leading to the development of cardiovascular or metabolic disorders [12]. Notably, moderate to severe OSA increases the risk of severe cardiovascular events such as stroke, heart attack, and heart failure (HF) [13]. One of the ways OSA can cause CVD is by inducing inflammation in the body. Studies have shown that OSA can result in systemic and local inflammation in patients. This inflammation can trigger the impairment of vascular endothelial cells and further modify the structure and function of vessels, leading to endothelial dysfunction. Endothelial dysfunction is a key factor in the development of various end-organ morbidities, such as CVD and metabolic dysfunction [12]. Also, in individuals with type 2 diabetes mellitus (T2DM), OSA is associated with a higher risk of retinopathy, dementia, chronic kidney disease (CKD), and decreased renal function [14,15,16]. Moreover, recent studies have found that nearly half of the people with diabetes had OSA, with a bidirectional association between OSA and diabetes [17,18,19]. Hence, it is of paramount importance to understand the association between OSA, diabetes, and CVD. Although individual database studies have been conducted globally [20,21,22,23,24], a comprehensive review is warranted to understand these associations. This systematic review aims to comprehensively elucidate the potential relationship between CVD and OSA in individuals with diabetes by examining and synthesizing all available evidence in this domain.

## 2. Methods

The systematic review followed the Preferred Reporting Items for Systematic Reviews and Meta-Analyses (PRISMA) guidelines and the MOOSE (Meta-analyses Of Observational Studies in Epidemiology) checklist throughout the process (Supplementary Material, Tables S1 and S2) [25,26]. The study protocol was registered with PROSPERO and was assigned the registration number CRD42023404126.

### 2.1. Inclusion and Exclusion Criteria

This systematic review considered observational studies to be eligible for inclusion. Original studies reporting cardiovascular events related to OSA in populations with type-1 and type-2 diabetes were included. This review excluded trials, protocols, narrative reviews, editorials, unpublished reports, clinical case reports, abstracts, and commentaries. Only preprints and published articles in English were considered. The study population included diabetic patients with OSA. Cross-sectional, cohort, case–control studies with prospective or retrospective designs were eligible if they reported any CVD events in OSA (disease) and non-OSA (control) groups in the diabetic population. Studies without a non-OSA control group and those reporting only cardiovascular risk factors were excluded. There were not any restrictions regarding the country or research environment (such as secondary care, primary care, or outpatients) (Table S3).

### 2.2. Search Strategy and Screening

On 15 July 2023, to find the relevant studies, a thorough literature search was carried out across several databases, including PubMed, EBSCO, Scopus, ProQuest, and Web of Science. The search strategy employed various keywords such as “OSA” OR “sleep apnea” OR “obstructive sleep apnea” AND “stroke” OR “cardiovascular disease” OR “coronary artery disease” OR “heart failure” OR “cardiovascular mortality” OR “CHD” OR “CVD” OR “CAD” OR “cardiac*” OR “Arrhythmia” OR “MI” AND “DM” OR “diabetes” OR “NIDDM” OR “type-2 diabetes*” OR “T2DM” OR “type 2 diabetes*” OR “T1DM”. The search was limited to English-language publications without year restrictions (Table S4). The search results were exported to Mendeley, and duplicates were eliminated. Rayyan was used to perform primary screening. Four investigators (G.B., B.K.P., M.S., and P.S.) separately screened the retrieved records by reading the titles and abstracts to eliminate irrelevant studies. Full-text readings of all the studies in the primary screening were carried out to ascertain eligibility. In cases of disagreement, three additional senior authors served as reviewers to resolve discrepancies. The reference lists of the included studies were thoroughly searched manually to identify any additional studies that might not have been previously identified in the initial search. In cases where duplicate reports from the same study or cohort were found, we made the decision to include the publication that offered the largest number of cases or the entire cohort.

### 2.3. Data Extraction and Quality Assessment

The eligible studies were subjected to data extraction by four researchers (G.B., B.K.P., M.S., and P.S.) separately. The name of the author, study design, country of research, year of publication, number of OSA and non-OSA patients, type of diabetes, number of participants included in the study, number of cardiovascular events in non-OSA as well as OSA groups, and the type of CVDs were extracted from the included studies in the data extraction process. The Newcastle–Ottawa Scale was used for critical appraisal of the studies in terms of quality and bias. The studies were scored based on three aspects: selection (0–4), comparability (0–2), and outcomes (0–3). The scoring system employed in this study demonstrated that a higher score indicated superior quality and a reduced risk of bias.

### 2.4. Statistical Analysis

In this study, we utilized R Version 4.3.1, a software package provided by the R Foundation, to perform all statistical analyses. Statistical analysis was performed by two of the authors (B.K.P. and J.J.B.) The inclusion criteria for the selected studies were based on reports of the dichotomous variable of CVD presence or absence in OSA and non-OSA groups within the diabetic population, along with the total number of patients in both groups. A pooled effect size was calculated using the risk ratio (RR) for CVD events that occurred in non-OSA and OSA patients in the diabetic population, and the Mantel–Haenszel (MH) model with random effects was utilized to generate the effect size with a 95% confidence interval (CI) and corresponding *p*-values. To assess heterogeneity across the studies, we employed I^2^ statistics and corresponding *p*-values, classifying heterogeneity as high (I^2^ > 50%), medium (I^2^ = 26–50%), and low (I^2^ < 25%). Additionally, we utilized the Paule–Mandel estimator for Tau^2^. Furthermore, to address heterogeneity through various statistical measures, we computed the 95% prediction interval for the pooled result. This interval provides an estimate of the range within which the true effects are expected for 95% of similar studies that may be conducted in the future [27]. To depict meta-analysis summaries, a forest plot was generated. We performed sensitivity analyses by omitting one study at a time to find the impact of individual studies on the pooled summary estimate. The publication bias was assessed by visually inspecting the funnel plot. Meta-regression was performed to evaluate the effect of variables on the overall effect size.

## 3. Results

### 3.1. Literature Search

**Figure 1:**
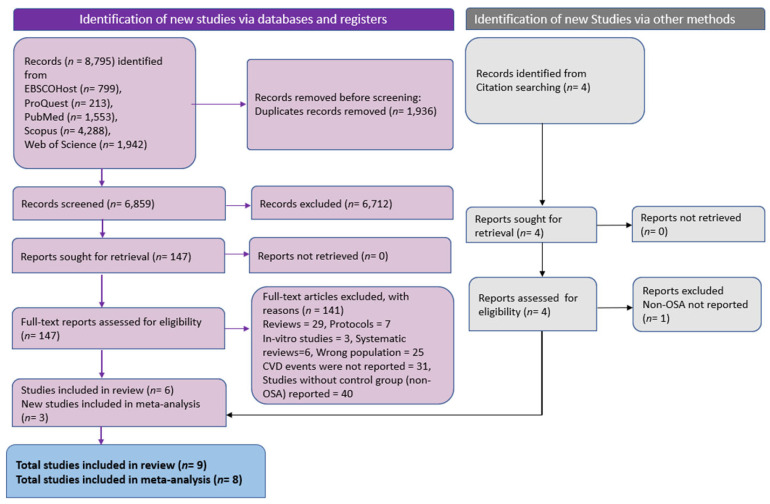
PRISMA flowchart depicting literature search and screening process (*n* indicates number of studies).

The process of conducting a systematic literature search and selecting relevant studies is presented in Figure 1. In the primary literature search, we found 8795 articles from multiple databases. Among these, 1936 studies were recognized as duplicates and omitted. Following the removal of duplicates, 6859 records underwent primary screening through title and abstract analysis. Of these, 6712 studies were deemed irrelevant and excluded, leaving 147 for full-text review. After thoroughly examining these studies, 141 articles were excluded based on predetermined criteria. Of these, 29 were narrative reviews, 6 were systematic reviewers, 7 were protocols, 25 had incorrect populations, 31 did not report the number of CVD events, 3 were in vitro studies, and 40 were without a control group. To identify additional studies, cross-reference checking was performed. In total, nine studies met the inclusion criteria, of which eight were eligible for meta-analysis. Six studies were obtained through the implemented search strategy, while the remaining three were identified through other methods.

### 3.2. Characteristics of Studies

The key characteristics of the studies that met the inclusion criteria for this systematic review and meta-analysis are summarized in Table 1. The nine included studies encompassed 17,796 diabetic patients, 4968 with OSA and 12,828 as non-OSA controls. Two studies were conducted in Singapore, two in the UK, and one each in China, France, Finland, and the United States. All the studies were conducted with type-2 diabetes patients except one study in which type-1 diabetics were included as participants [28]. Most studies reported coronary heart disease (CHD), stroke, and MI. Out of a score of 9, four studies scored 8, three scored 7, and two scored 6 on the Newcastle–Ottawa Scale. The studies were of moderate quality overall (Table S5).

### 3.3. Association of OSA with CVD

Data from eight independent studies were analyzed using a random-effects model. The findings revealed varying RR values for different CVD conditions: for stroke, RR was 0.69 (95% CI: 0.30–1.59) with a *p*-value of 0.30; for ischemic heart disease (IHD), RR was 1.34 (95% CI: 1.21–1.43) with a *p*-value of 0.01; RR for atrial fibrillation (AF) was 1.29 (95% CI: 0.01–125.64) with a *p*-value of 0.60; for peripheral vascular disease (PVD), RR was 1.82 (95% CI: 0.48–6.96) with a *p*-value of 0.19; and myocardial infarction (MI) yielded an RR of 1.24 (95% CI: 0.50–3.10) with a *p*-value of 0.512.

Only single studies reported outcomes such as HF, acute coronary syndrome (ACS), CHD, and major adverse cardiovascular and cerebrovascular events (MACCE) separately. Therefore, meta-analysis was not possible for these separate CVD outcomes. HF exhibited a RR of 2.54 (95% CI: 2.20–2.93) with a significant *p*-value (<0.001); acute coronary syndrome (ACS) displayed a substantial RR of 11.61 (95% CI: 1.57–85.70) with a *p*-value of 0.01; RR for developing CHD was 1.42 (95% CI: 0.57–3.54) with a *p*-value of 0.45; and the RR for developing major adverse cardiovascular and cerebrovascular event (MACCE) was 3.15 (95% CI: 1.54–6.42) with a *p*-value of 0.001.

**Figure 2:**
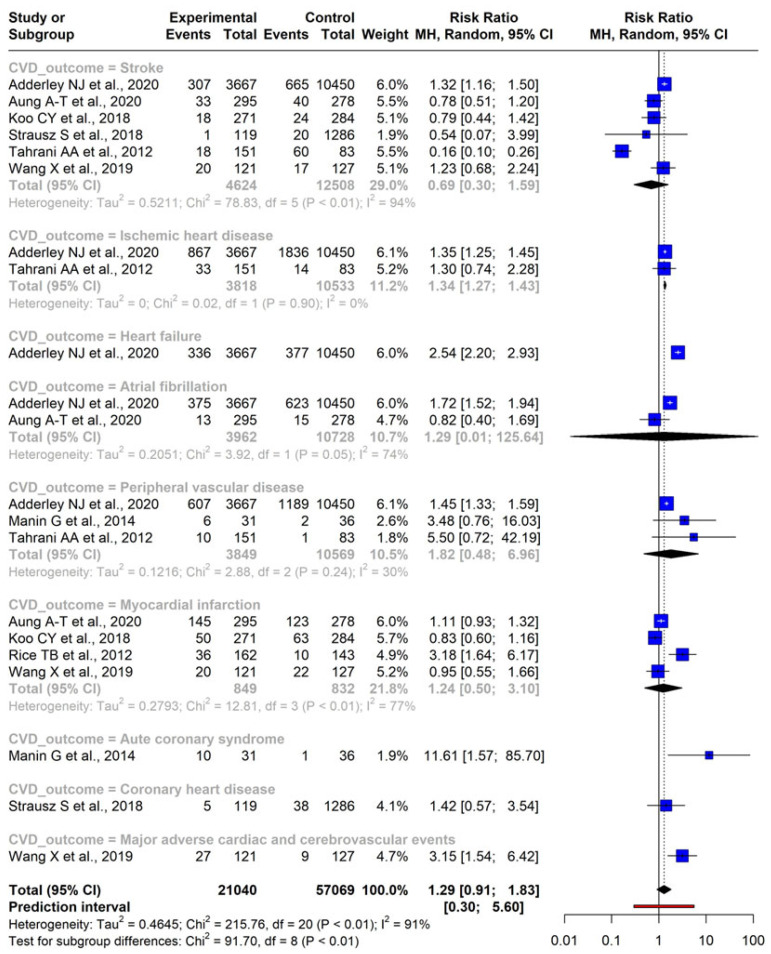
Forest plot indicating the association between OSA and CVD outcomes [14,20,21,22,23,29,30,31].

The overall pooled result for developing all CVD combined was an RR of 1.29 (95% CI: 0.91–1.83), with a *p*-value of 0.1478, and a 95% prediction interval ranging from 0.30 to 5.60. It is essential to note that significant heterogeneity was observed among the included studies (I^2^ = 91%). The forest plot showcasing the various CVD conditions and the combined RR estimate is given in Figure 2. One study did not report the number of CVD events and was omitted from meta-analysis [32]. This study reported that the combined presence of OSA and DM was found to be independently associated with a higher risk of incident CVD mortality (HR 2.37, CI 1.16–4.82, *p* = 0.02) and an increased prevalence of CHD (OR 3.44, CI 1.73–5.59, *p* < 0.01).

### 3.4. Meta-Regression

**Figure 3:**
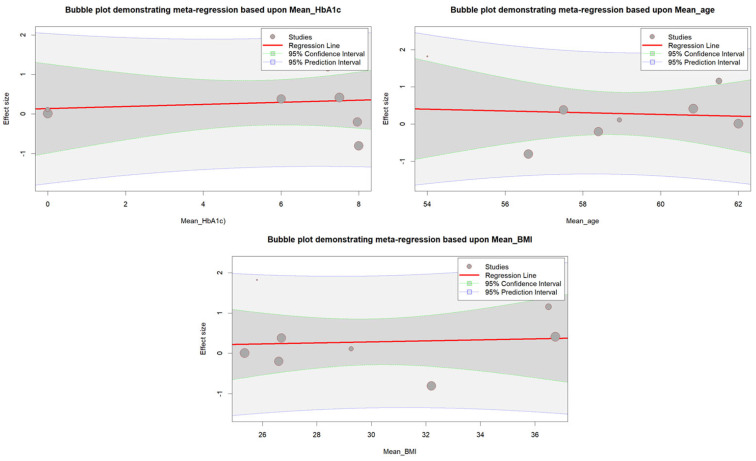
Bubble plot illustrating the meta-regression analysis examining the association between mean age, mean BMI, and mean HbA1c with effect sizes.

Meta-regression was conducted to examine the potential impact of mean HbA1c, BMI, and age on the results. However, none of the variables were found to be significantly associated with the results (*p* = 0.8451 for age, *p* = 0.8484 for BMI, and *p* = 0.7680 for HbA1c). Figure 3 shows a bubble plot that displays the relationship between the different variables with the result.

### 3.5. Publication Bias and Sensitivity Analysis

This study utilized a funnel plot to graphically present publication bias, and a considerable publication bias was observed (Figure S1). Using a random-effect model, the leave-one-out analysis systematically omitted one study at a time based on heterogeneity (I^2^) and effect size. The results showed no significant changes in the overall pooled RR, which ranged from 1.13 to 1.52, or in the level of heterogeneity, which ranged from 91% to 97% (Figure S2).

## 4. Discussion

This study, encompassing a systematic review and meta-analysis, is the first to examine the link between CVD and OSA in individuals with diabetes exclusively. An RR of 1.29 (CI: 95%, 0.91–1.83) for CVD was observed in diabetics with OSA; however, it was not significant compared to non-OSA counterparts. However, individual CVD outcomes, such as ACS, HF, IHD, and MACCE, were found to be significantly associated with OSA in diabetic patients. CVD outcomes such as stroke, MI, PVD, CHD, and AF were not significantly associated with OSA in our analysis.

Although only eight studies that reported the association of CVD in the diabetic OSA population were available, we found high heterogeneity across these studies, which sensitivity analysis and meta-regression failed to resolve. This high heterogeneity could be the reason for the varying results for different CVD outcomes. The characteristics of the participants in the studies were also widely varied, which may have contributed to heterogeneity. A previous systematic review on post-percutaneous coronary intervention (PCI) in a diabetic population with OSA reported a significant increase in MACCEs and all-cause mortality [33]. Several meta-analyses have reported OSA as an independent risk factor for CVD [28,34,35,36,37]. Diabetes is a classical risk factor for CVD, and the presence of OSA with diabetes may accelerate the emergence of adverse CVD outcomes. Our results showed a cumulative effect of OSA and diabetes on cardiovascular outcomes.

OSA is a prevalent sleep disorder characterized by repeated upper airway obstruction during sleep. This results in reduced oxygen intake by the lungs, increased carbon dioxide levels in the body, and frequent sleep pattern disruptions. It has been hypothesized to elevate the risk of various CVDs, including stroke, in affected individuals [38]. Although the specific biological processes that underlie the association between CVD and OSA are not entirely understood, some mechanisms have been postulated, such as changes in chest pressure, heightened activity in the sympathetic nervous system, inflammation, and oxidative stress in blood vessels due to cycles of oxygen deprivation and restoration during sleep [38]. The sympathetic nervous system is hypothesized to be activated due to heightened sympathetic drive induced by repeated episodes of apnea and hypopnea, resulting in lower amounts of oxygen in the blood and increased carbon dioxide levels. Recurrent upper airway collapse during sleep, which results in the partial or total cessation of airflow despite continued respiratory effort, is a defining feature of OSA. This can increase respiratory effort against the blocked upper airway, leading to negative intrathoracic pressure. Research has suggested that OSA may be linked to higher levels of inflammatory cytokines and metabolic dysregulation, which may contribute to the development of atherosclerosis. However, due to the complex and diverse nature of OSA, the precise mechanisms that link it to CVD remain somewhat unclear [39].

The presence of diabetes with OSA enhances CVD risk. This is possible because OSA is associated with repetitive episodes of hypoxia–reoxygenation, triggering a cascade of metabolic and inflammatory changes that exacerbate pre-existing metabolic and cardiovascular risk factors in diabetes [11]. One potential mechanism involves insulin resistance, which is common in diabetes and known to promote inflammation and oxidative stress [40]. OSA is linked with impaired glucose metabolism and increased insulin resistance, leading to further inflammation, endothelial dysfunction, and the development of atherosclerosis and CVDs [41]. Another potential mechanism involves activating the renin–angiotensin–aldosterone system (RAAS), associated with maintaining blood pressure (BP). OSA activates the RAAS system, leading to an increase in BP and causing volume overload, contributing to the development of hypertension and CVDs [42]. Additionally, OSA is associated with increased sympathetic nervous system activity, which can further exacerbate hypertension and CVD risk in diabetes. Sympathetic activation can increase heart rate and vasoconstriction, leading to arrhythmias [43]. Overall, the mechanisms linking CVD and OSA in diabetics are likely multifactorial and involve a complex interplay between metabolic and inflammatory pathways.

All the included studies in this review were conducted among the type-2 diabetic population, except one in which type-1 diabetic participants were included [30]. Several studies in our meta-analysis directly investigated the relationship between OSA, diabetes, and CVD. Also, few studies explored the relationship with individual CVDs. For example, a cohort study conducted by Adderly et al. revealed that people with DM who developed OSA were at a greater risk of developing CVDs by over 50% [21]. Individuals with diabetes who developed OSA face a greater risk of peripheral neuropathy (PN), AF, diabetic foot disease (DFD), CKD, and all-cause mortality when compared with diabetics without OSA, even after adjusting for potential confounding factors [21]. This study also found that the newly diagnosed DM and OSA patients were at high risk of composite PN, DFD, CVD, and albuminuria [21]. However, the secondary analysis found no significant association between the individual factors of composite CKD, AF, CVD outcome, and all-cause death. This difference may be due to the shorter diabetes duration or better prevention in the OSA group before diabetes diagnosis. Similarly, another observational study found that individuals undergoing coronary artery bypass grafting (CABG) had a relatively higher prevalence of developing OSA and DM [20].The study also found that individuals with both OSA and DM had an elevated probability of emerging MACCEs and were more likely to be hospitalized for HF [20]. Findings further demonstrated that the presence of OSA and DM is independently related to the risk of MACCEs and hospitalization for HF following CABG, even after controlling for left ventricular ejection fraction and medication use [20]. However, it should be mentioned that this study had a few limitations, such as the possibility of unknown or unmeasured confounding variables, and the generalizability of the findings to emergency CABG or major non-cardiac-related surgeries may be limited. Also, the predominantly Asian study population had a limited representation of women subjects [20].

A prospective cohort study by Wang et al. demonstrated a link between OSA and DM, where patients with both conditions were at greater risk of MACCE following an ACS episode, compared to those with DM alone [22]. Interestingly, the prevalence of MACCE among non-DM patients was comparable between individuals with and without OSA. The high risk of adverse effects was mainly observed in patients with OSA and poor glucose control, which was further exacerbated in the presence of prolonged hypoxia, particularly if accompanied by DM [22]. A cross-sectional study by Rice et al. found a positive association between the apnea-hypopnea index (AHI) and stroke risk among obese individuals with type 2 DM, indicating that moderate and severe OSA can elevate the risk of stroke [23]. Notably, no significant association was observed between OSA and baseline CHD. These findings also suggest that identifying and addressing OSA may be crucial for mitigating stroke risk among obese individuals with DM [20,21,22,23].

These findings highlight the importance of considering the link between OSA and diabetes and not overlooking its potential impact on the occurrence of cardiovascular diseases. It is crucial to suggest regular screening for CVDs in patients with both OSA and diabetes to catch any issues early and manage them appropriately. Clinicians need to stay vigilant and well-informed about the potential effects of OSA when it coexists with diabetes, as it can lead to various negative cardiovascular outcomes. However, more research is needed to strengthen our understanding of this connection and how it affects clinical practice.

The results of this systematic review were derived from articles in English only, potentially excluding relevant publications in other languages. In addition, due to the relative novelty of the topic, the number of studies found to be eligible for analysis was limited. Despite our efforts to address heterogeneity through sensitivity analysis and meta-regression, the results were not fully satisfactory. Moreover, some studies in our analysis focused on patients who had already undergone interventions such as CABG and cardiac revascularization, which may have influenced our findings. Additionally, while most studies directly assessed the relationship between OSA and CVD in diabetics, some studies did not consider other cardiovascular risk factors that may be prevalent in study participants. Finally, several studies in our analysis had small sample sizes, which may limit the generalizability of our findings. Owing to the high heterogeneity across the available studies, further research is warranted to assess the association between OSA and CVD outcomes in diabetics. Future studies with larger sample sizes and more standardized protocols will improve the results’ reliability and generalizability and enable scholars to focus on identifying effective interventions to prevent and manage OSA in patients with diabetes to reduce the risk of CVDs.

## 5. Conclusions

In conclusion, this study emphasizes the significance of acknowledging the association between OSA and diabetes and its potential influence on CVDs. Clinicians must remain vigilant and informed about the possible cardiovascular risks when OSA coexists with diabetes. The early detection and treatment of OSA in patients with diabetes may lower healthcare utilization and CVD risk in this population. The results of our study emphasize the necessity for future research to gain a more comprehensive understanding of the intricate connection between OSA, diabetes, and CVD, thereby establishing efficient interventions for the prevention and management of these conditions in clinical practice.

## Figures and Tables

**Table 1:** Characteristics of included studies.

Author	Study Design	Country, Year	Age (Mean)	Male %	Type of Diabetes	Mean Follow-Up Period (Years)	BMI (Mean)	HbA1c (Mean)	Total OSA	No. of CVD Events in OSA	Total Non-OSA	No. of CVD Events in non OSA	Total Sample Size	Type of CVD Outcome
Adderley NJ et al. [21]	Retrospective cohort	UK, 2020	60.84	75.00	T2DM	3	36.75	7.50	3667	2492	10,450	4690	14,117	Stroke/TIA, IHD, HF, AF, PVD
Aung A-T et al. [20]	Prospective, observational	Singapore, 2020	62.00	83.30	T2DM	2.1	25.35	N/A	295	191	278	178	573	Stroke/TIA, MI, AF
Koo CY et al. [29]	Prospective, observational	Singapore, 2018	58.4	85.20	T2DM	1.9	26.60	7.96	271	68	284	87	555	MI, Stroke
Manin G et al. [30]	Cross-sectional	France, 2014	54.00	60.00	T1DM	N/A	25.80	7.55	31	16	36	3	67	ACS, PVD
Rice TB et al. [23]	Cross-sectional	USA, 2012	61.50	40.00	T2DM	N/A	36.50	7.20	162	36	143	10	305	MI
Strausz S et al. [31]	Prospective cohort	Finland, 2018	58.94	51.20	T2DM	12.9	29.26	N/A	119	6	1286	58	1405	CHD, Stroke/TIA
Tahrani AA [14]	Cross-sectional	UK, 2012	56.6	57.60	T2DM	N/A	32.2	8	151	61	83	75	234	Stroke/TIA, IHD, PVD
Wang X et al. [22]	Prospective cohort	China, 2019	57.50	82.60	T2DM	2	26.70	6	121	67	127	48	248	Stroke, MI, MACCE
Labarca et al. [32]	Prospective cohort	Chile, 2021	59.4	67.81	T2DM	5	32.3	N/A	151	N/A	141	N/A	292	CV mortality, stroke, CHD

ACS: acute coronary syndrome; AF: atrial fibrillation; CHD: coronary heart disease; CVD: cardiovascular diseases; HF: heart failure; OSA: obstructive sleep apnea; IHD: ischemic heart disease; MACCEs: major adverse cardiovascular and cerebrovascular event; MI: myocardial infraction, PVD: peripheral vascular disease; T1DM: type-1 diabetes mellitus; T2DM: type-2 diabetes mellitus; TIA: transient ischemic attack; UK: United Kingdon; USA: United States of America; N/A: Not available.

## Data Availability

Documents containing all extracted data are available in the manuscript and the accompanying Supplementary Material.

## Supplementary Materials

The following supporting information can be downloaded at: https://www.mdpi.com/article/10.3390/diseases11030103/s1, Table S1: PRISMA Checklist 2020, Table S2: MOOSE (Meta-analyses Of Observational Studies in Epidemiology) Checklist, Table S3: Inclusion and Exclusion criteria, Table S4: The adjusted search terms as per searched electronic databases [as of 15 July 2023], Table S5: Newcastle-Ottawa Scale for assessing the quality and Figure S1: Funnel plot for the assessment of publication bias, Figure S2: Leave-One-Out Meta-Analysis Results.
