# Supplementary material for: Cardiovascular Disease Outcomes Associated with Obstructive Sleep Apnea in Diabetics: A Systematic Review and Meta-Analysis
Source: Diseases. 2023 Aug 7;11(3):103. doi: 10.3390/diseases11030103 (PMC10443251; doi:10.3390/diseases11030103)
Supplement: Supplementary file 1 [file diseases-11-00103-s001.zip › diseases-2473580-supplementary.pdf]

Supplementary Materials

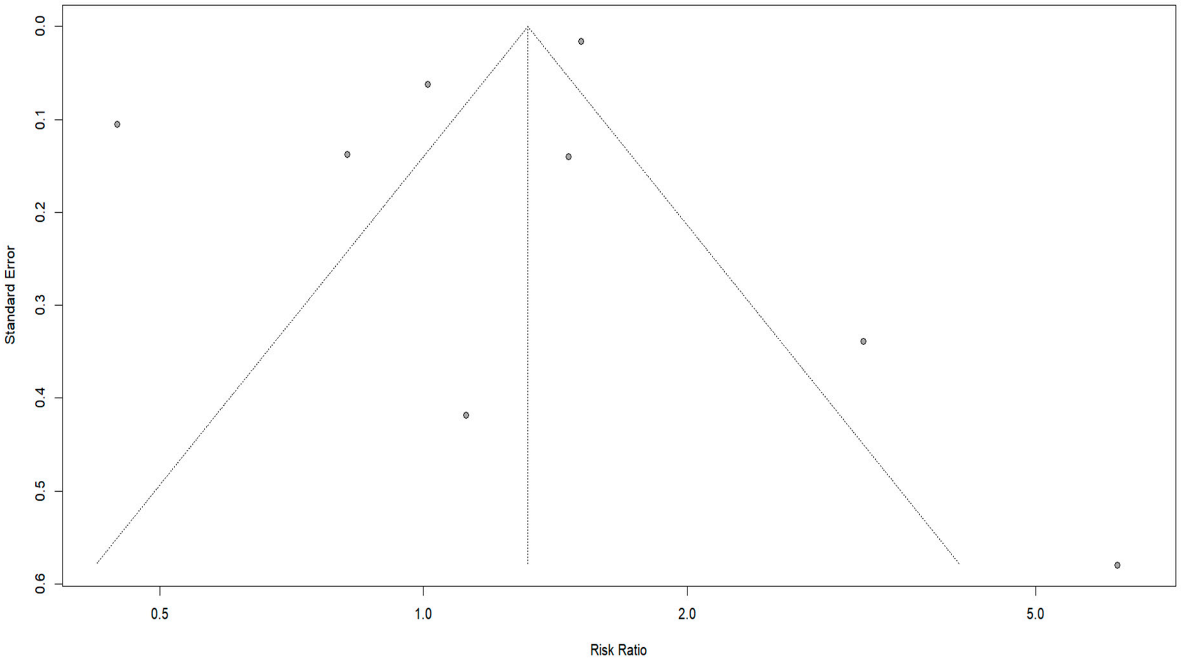

Figure S1. Funnel plot for the assessment of publication bias.

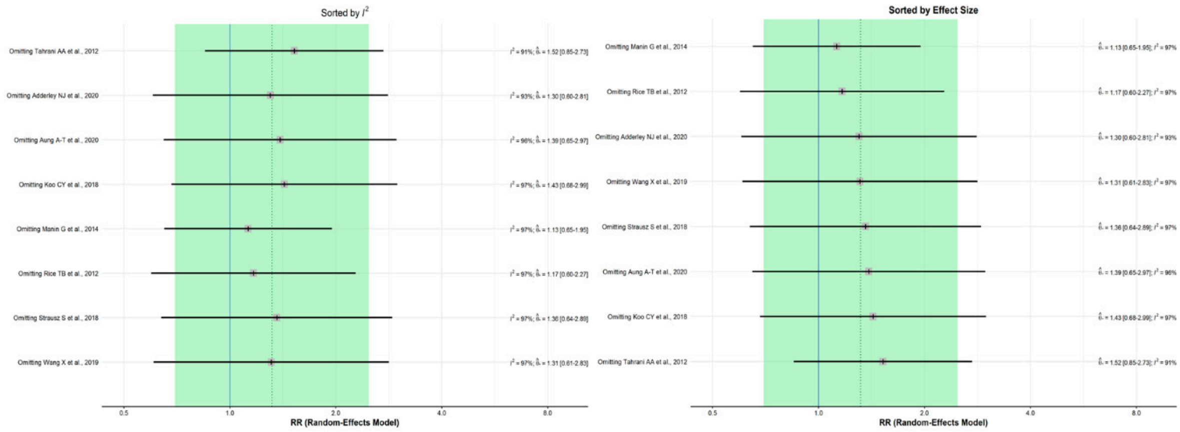

Figure S2. Leave-One-Out Meta-Analysis Results.

Table S1. PRISMA Checklist 2020.

Section and Topic	Item #	Checklist item (Cardiovascular outcomes associated with obstructive sleep apnea in diabetic population: A systematic review and Meta-analysis)	Location where item is reported
**TITLE**			
Title	1	Identify the report as a systematic review.	1
		**ABSTRACT**	
Abstract	2	See the PRISMA 2020 for Abstracts checklist. (Made as per the Journal guidelines)	2
		**INTRODUCTION**	
Rationale	3	Describe the rationale for the review in the context of existing knowledge.	3
Objectives	4	Provide an explicit statement of the objective(s) or question(s) the review addresses.	3, Table S2
**METHODS**			
Eligibility criteria	5	Specify the inclusion and exclusion criteria for the review and how studies were grouped for the syntheses.	3
Information sources	6	Specify all databases, registers, websites, organisations, reference lists and other sources searched or consulted to identify studies. Specify the date when each source was last searched or consulted.	3, 4 & Table S3
Search strategy	7	Present the full search strategies for all databases, registers and websites, including any filters and limits used.	Table S3
Selection process	8	Specify the methods used to decide whether a study met the inclusion criteria of the review, including how many reviewers screened each record and each report retrieved, whether they worked independently, and if applicable, details of automation tools used in the process.	3,4
Data collection process	9	Specify the methods used to collect data from reports, including how many reviewers collected data from each report, whether they worked independently, any processes for obtaining or confirming data from study investigators, and if applicable, details of automation tools used in the process.	4
Data items	10a	List and define all outcomes for which data were sought. Specify whether all results that were compatible with each outcome domain in each study were sought (e.g. for all measures, time points, analyses), and if not, the methods used to decide which results to collect.	10, Table 1
	10b	List and define all other variables for which data were sought (e.g. participant and intervention characteristics, funding sources). Describe any assumptions made about any missing or unclear information.	10, Table 1
Study risk of bias assessment	11	Specify the methods used to assess risk of bias in the included studies, including details of the tool(s) used, how many reviewers assessed each study and whether they worked independently, and if applicable, details of automation tools used in the process.	5, Table S4
Effect measures	12	Specify for each outcome the effect measure(s) (e.g., risk ratio, mean difference) used in the synthesis or presentation of results.	4
Synthesis methods	13a	Describe the processes used to decide which studies were eligible for each synthesis (e.g., tabulating the study intervention characteristics and comparing against the planned groups for each synthesis (item #5)).	10, Table 1
	13b	Describe any methods required to prepare the data for presentation or synthesis, such as handling of missing summary statistics, or data conversions.	NA
	13c	Describe any methods used to tabulate or visually display results of individual studies and syntheses.	Figure 1
	13d	Describe any methods used to synthesize results and provide a rationale for the choice(s). If meta-analysis was performed, describe the model(s), method(s) to identify the presence and extent of statistical heterogeneity, and software package(s) used.	4
	13e	Describe any methods used to explore possible causes of heterogeneity among study results (e.g., subgroup analysis, meta-regression).	4
	13f	Describe any sensitivity analyses conducted to assess robustness of the synthesized results.	4

Section and Topic	Item #	Checklist item (Cardiovascular outcomes associated with obstructive sleep apnea in diabetic population: A systematic review and Meta-analysis)	Location where item is reported
Reporting bias assessment	14	Describe any methods used to assess risk of bias due to missing results in a synthesis (arising from reporting biases).	NA
Certainty assessment	15	Describe any methods used to assess certainty (or confidence) in the body of evidence for an outcome.	NA
**RESULTS**			
Study selection	16a	Describe the results of the search and selection process, from the number of records identified in the search to the number of studies included in the review, ideally using a flow diagram.	5
	16b	Cite studies that might appear to meet the inclusion criteria, but which were excluded, and explain why they were excluded.	Figure 1
Study characteristics	17	Cite each included study and present its characteristics.	10, Table 1
Risk of bias in studies	18	Present assessments of risk of bias for each included study.	5, Table S4
Results of individual studies	19	For all outcomes, present, for each study: (a) summary statistics for each group (where appropriate) and (b) an effect estimates and its precision (e.g., confidence/credible interval), ideally using structured tables or plots.	Table 1, Figure 2
Results of syntheses	20a	For each synthesis, briefly summarise the characteristics and risk of bias among contributing studies.	5
	20b	Present results of all statistical syntheses conducted. If meta-analysis was done, present for each the summary estimate and its precision (e.g., confidence/credible interval) and measures of statistical heterogeneity. If comparing groups, describe the direction of the effect.	5, 6
	20c	Present results of all investigations of possible causes of heterogeneity among study results.	6, Figure S2
	20d	Present results of all sensitivity analyses conducted to assess the robustness of the synthesized results.	Figure S2
Reporting biases	21	Present assessments of risk of bias due to missing results (arising from reporting biases) for each synthesis assessed.	NA
Certainty of evidence	22	Present assessments of certainty (or confidence) in the body of evidence for each outcome assessed.	NA
**DISCUSSION**			
Discussion	23a	Provide a general interpretation of the results in the context of other evidence.	6, 7
	23b	Discuss any limitations of the evidence included in the review.	8
	23c	Discuss any limitations of the review processes used.	8
	23d	Discuss implications of the results for practice, policy, and future research.	8
**OTHER INFORMATION**			
Registration and protocol	24a	Provide registration information for the review, including register name and registration number, or state that the review was not registered.	3
	24b	Indicate where the review protocol can be accessed, or state that a protocol was not prepared.	3
	24c	Describe and explain any amendments to information provided at registration or in the protocol.	NA
Support	25	Describe sources of financial or non-financial support for the review, and the role of the funders or sponsors in the review.	9
Competing interests	26	Declare any competing interests of review authors.	9
Availability of data, code and other materials	27	Report which of the following are publicly available and where they can be found template data collection forms; data extracted from included studies; data used for all analyses; analytic code; any other materials used in the review.	NA

Table S2. MOOSE (Meta-analyses Of Observational Studies in Epidemiology) Checklist.

Reporting Criteria	Reported (Yes/No)	Reported on Page No.
**Reporting of Background**	Yes	2
Problem definition	Yes	2
Hypothesis statement	Yes	2
Description of Study Outcome(s)	Yes	2
Type of exposure or intervention used	Yes	2
Type of study design used	Yes	2,3
Study population	Yes	2
**Reporting of Search Strategy**		
Qualifications of searchers (eg, librarians and investigators)	Yes	3
Search strategy, including time period included in the synthesis and keywords	Yes	3
Effort to include all available studies, including contact with authors	Yes	3
Databases and registries searched	Yes	3
Search software used, name and version, including special features used (eg, explosion)	No	
Use of hand searching (eg, reference lists of obtained articles)	Yes	4
List of citations located and those excluded, including justification	Yes	4
Method for addressing articles published in languages other than English	Yes	4
Method of handling abstracts and unpublished studies	No	
Description of any contact with authors	No	
**Reporting of Methods**		
Description of relevance or appropriateness of studies assembled for assessing the hypothesis to be tested	Yes	2
Rationale for the selection and coding of data (eg, sound clinical principles or convenience)	Yes	2
Documentation of how data were classified and coded (eg, multiple raters, blinding, and interrater reliability)	No	
Assessment of confounding (eg, comparability of cases and controls in studies where appropriate	Yes	3
**Reporting Criteria**	**Reported (Yes/No)**	**Reported on Page No.**
Assessment of study quality, including blinding of quality assessors; stratification or regression on possible predictors of study results	Yes	3
Assessment of heterogeneity	Yes	3
Description of statistical methods (eg, complete description of fixed or random effects models, justification of whether the chosen models account for predictors of study results, dose-response models,	Yes	3

or cumulative meta-analysis) in sufficient detail to be replicated		
Provision of appropriate tables and graphics	No	
**Reporting of Results**		
Table giving descriptive information for each study included	Yes	6, Table 1
Results of sensitivity testing (eg, subgroup analysis)	Yes	8
Indication of statistical uncertainty of findings	Yes	8
**Reporting of Discussion**		
Quantitative assessment of bias (eg, publication bias)	Yes	3
Justification for exclusion (eg, exclusion of non–English-language citations)	Yes	2
Assessment of quality of included studies	Yes	3
**Reporting of Conclusions**		
Consideration of alternative explanations for observed results	Yes	11
Generalization of the conclusions (ie, appropriate for the data presented and within the domain of the literature review)	Yes	11
Guidelines for future research	Yes	11
Disclosure of funding source	Yes	12

Table S3. Inclusion and Exclusion criteria.

Research Question: What is the association between obstructive sleep apnea (OSA) and cardiovascular diseases (CVDs) in diabetic patients?

	Inclusion	Exclusion
**Participants**	OSA-confirmed patients with diabetes<br>All gender<br>All age groups	Non-diabetic and pre-diabetic
**Disease**	Any type of diabetes (Type-1 and Type-2)<br>Any grade of OSA (Grade-1, Grade-2, Grade-3, Grade-4, Grade-5)	
**Comparator**	Diabetic patients without OSA	
**Outcome**	Cardiovascular diseases: coronary heart disease, stroke, myocardial infraction, cardiovascular mortality, heart failure, coronary artery disease, cerebrovascular accident, major adverse cardiovascular and cerebrovascular event	Cardiovascular risk factors like hypertension, atherosclerosis, elevated cardiac biomarkers, all-cause mortality
**Study Designs**	Cross-sectional studies, cohort studies, case-control studies	Qualitative, policy, opinion, case studies, case-reports, prevalence studies, case series, surveys
	Geography-Global level<br>Date of Search- Publish till 1st of March 2023<br>English Language<br>Human studies	
	Published articles and preprints data	

Table S4. The adjusted search terms as per searched electronic databases [as of 15.07.2023].

Database	No	Search Query	Results
**Web of Science**			
	**#1**	**"OSA" OR "obstructive sleep apnea" OR "sleep apnea" OR "Sleep-disordered breathing" (Topic)**	**89,992**
	**#2**	**"T2DM" OR "diabetes" OR "NIDDM" OR "type-2 diabet*" OR "mellitus" OR "DM" OR "type II diabet*" OR "type 2 diabet*" OR "diabetic*" OR "T1DM" (Topic)**	**1,041,030**
	**#3**	**"heart disease" OR "vascular diseases" OR "stroke" OR "cerebrovascular accident" OR "sudden death" OR "cardiac arrest" OR "cardiovascular disease" OR "coronary artery disease" OR "heart failure" OR "cardiovascular mortality" OR "coronary death" OR "CHD" OR "CVD" OR "cardiac death" OR "myocardial infarction" OR "angina" OR "CAD" OR cardiac* OR myocardial* OR "Aortic disease" OR "congestive heart failure" OR cardio* OR "ischemic heart disease" OR "atherosclerosis" OR "Heart muscle disease" OR "Deep vein thrombosis" OR "pulmonary embolism" OR "Pericardial disease" OR "Rheumatic heart disease" OR "MI" OR "CVA" OR "heart" OR "re-infarction" OR re-vascularization OR "cardiac mortality" OR "Acute coronary syndrome" OR "percutaneous coronary intervention" OR "arrhythmia" OR "atrial fibrillation" OR "pericarditis" OR "arrhythmia" (Topic)**	**3,293,220**
	**#4**	**Review OR rat OR rats OR animals OR animal OR in-vitro OR rabbit OR rabbits (Topic)**	**7,899,707**
	**#5**	#1 AND #2 AND #3 NOT #4	**2,041**
	**#6**	#1 AND #2 AND #3 NOT #4 **and English (Languages)**	**1,942**
**EBSCOHost-Academic Search Complete**			
	**#1**	AB "OSA" OR "obstructive sleep apnea" OR "sleep apnea" OR "Sleep-disordered breathing"	23,826
	**#2**	AB "T2DM" OR "diabetes" OR "NIDDM" OR "type-2 diabet*" OR "mellitus" OR "DM" OR "type II diabet*" OR "type 2 diabet*" OR "diabetic*" OR "T1DM"	485,790
	**#3**	AB **"heart disease" OR "vascular diseases" OR "stroke" OR "cerebrovascular accident" OR "sudden death" OR "cardiac arrest" OR "cardiovascular disease" OR "coronary artery disease" OR "heart failure" OR "cardiovascular mortality" OR "coronary death" OR "CHD" OR "CVD" OR "cardiac death" OR "myocardial infarction" OR "angina" OR "CAD" OR cardiac* OR myocardial* OR "Aortic disease" OR "congestive heart failure" OR cardio* OR "ischemic heart disease" OR "atherosclerosis" OR "Heart muscle disease" OR "Deep vein thrombosis" OR "pulmonary embolism" OR "Pericardial disease" OR "Rheumatic heart disease" OR "MI" OR "CVA" OR "heart" OR "re-infarction" OR re-vascularization OR "cardiac mortality" OR "Acute coronary syndrome" OR "percutaneous coronary intervention" OR "arrhythmia" OR "atrial fibrillation" OR "pericarditis" OR "arrhythmia"**	1,366,213

	#4	AB Review OR rat OR rats OR animals OR animal OR in-vitro OR rabbit OR rabbits	8,783,996
	#5	S1 AND S2 AND S3 NOT S4	843
	#6	S1 AND S2 AND S3 NOT S4 (Narrow by Language: - English)	799
**ProQuest**			
	#1	summary("OSA" OR "obstructive sleep apnea" OR "sleep apnea" OR "Sleep-disordered breathing")	5,244
	#2	summary("T2DM" OR "diabetes" OR "NIDDM" OR "type-2 diabet*" OR "mellitus" OR "DM" OR "type II diabet*" OR "type 2 diabet*" OR "diabetic*" OR "T1DM")	120,695
	#3	summary("heart disease" OR "vascular diseases" OR "stroke" OR "cerebrovascular accident" OR "sudden death" OR "cardiac arrest" OR "cardiovascular disease" OR "coronary artery disease" OR "heart failure" OR "cardiovascular mortality" OR "coronary death" OR "CHD" OR "CVD" OR "cardiac death" OR "myocardial infarction" OR "angina" OR "CAD" OR cardiac* OR myocardial* OR "Aortic disease" OR "congestive heart failure" OR cardio* OR "ischemic heart disease" OR "atherosclerosis" OR "Heart muscle disease" OR "Deep vein thrombosis" OR "pulmonary embolism" OR "Pericardial disease" OR "Rheumatic heart disease" OR "MI" OR "CVA" OR "heart" OR "re-infarction" OR re-vascularization OR "cardiac mortality" OR "Acute coronary syndrome" OR "percutaneous coronary intervention" OR "arrhythmia" OR "atrial fibrillation" OR "pericarditis" OR "arrhythmia")	230,408
	#4	summary(Review OR rat OR rats OR animals OR animal OR in-vitro OR rabbit OR rabbits)	793,164
	#5	1 AND 2 AND 3 NOT 4	215
	#6	1 AND 2 AND 3 NOT 4 (English)	213
**PubMed**			
	#1	"OSA"[Title/Abstract] OR "obstructive sleep apnea"[Title/Abstract] OR "sleep apnea"[Title/Abstract] OR "Sleep-disordered breathing"[Title/Abstract]	48,577
	#2	"T2DM"[Title/Abstract] OR "diabetes"[Title/Abstract] OR "NIDDM"[Title/Abstract] OR "type-2 diabet*"[Title/Abstract] OR "mellitus"[Title/Abstract] OR "DM"[Title/Abstract] OR "type II diabet*"[Title/Abstract] OR "type 2 diabet*"[Title/Abstract] OR "diabetic*"[Title/Abstract] OR "T1DM"[Title/Abstract]	809,830
	#3	"heart disease"[Title/Abstract] OR "vascular diseases"[Title/Abstract] OR "stroke"[Title/Abstract] OR "cerebrovascular accident"[Title/Abstract] OR "sudden death"[Title/Abstract] OR "cardiac arrest"[Title/Abstract] OR "cardiovascular disease"[Title/Abstract] OR "coronary artery disease"[Title/Abstract] OR "heart failure"[Title/Abstract] OR "cardiovascular mortality"[Title/Abstract] OR "coronary death"[Title/Abstract] OR "CHD"[Title/Abstract] OR "CVD"[Title/Abstract]	2,579,493

		OR "cardiac death"[Title/Abstract] OR "myocardial infarction"[Title/Abstract] OR "angina"[Title/Abstract] OR "CAD"[Title/Abstract] OR cardiac*[Title/Abstract] OR myocardial*[Title/Abstract] OR "Aortic disease"[Title/Abstract] OR "congestive heart failure"[Title/Abstract] OR cardio*[Title/Abstract] OR "ischemic heart disease"[Title/Abstract] OR "atherosclerosis"[Title/Abstract] OR "Heart muscle disease"[Title/Abstract] OR "Deep vein thrombosis"[Title/Abstract] OR "pulmonary embolism"[Title/Abstract] OR "Pericardial disease"[Title/Abstract] OR "Rheumatic heart disease"[Title/Abstract] OR "MI"[Title/Abstract] OR "CVA"[Title/Abstract] OR "heart"[Title/Abstract] OR "re-infarction"[Title/Abstract] OR re-vascularization[Title/Abstract] OR "cardiac mortality"[Title/Abstract] OR "Acute coronary syndrome"[Title/Abstract] OR "percutaneous coronary intervention"[Title/Abstract] OR "arrhythmia"[Title/Abstract] OR "atrial fibrillation"[Title/Abstract] OR "pericarditis"[Title/Abstract] OR "arrhythmia"[Title/Abstract]	
	**#4**	Review[Title/Abstract] OR rat[Title/Abstract] OR rats[Title/Abstract] OR animals[Title/Abstract] OR animal[Title/Abstract] OR in-vitro[Title/Abstract] OR rabbit[Title/Abstract] OR rabbits[Title/Abstract]	**5,790,977**
	**#5**	**((#1) AND (#2) AND (#3)) NOT (#4)**	**1,667**
	**#6**	**((#1) AND (#2) AND (#3)) NOT (#4) Filters: English**	**1,553**
**Scopus**			
	**#1**	**TITLE-ABS-KEY (** "osa" OR "obstructive sleep apnea" OR "sleep apnea" OR "sleep-disordered breathing" **)**	**130,038**
	**#2**	**TITLE-ABS-KEY (** "t2dm" OR "diabetes" OR "niddm" OR "type-2 diabet*" OR "mellitus" OR "dm" OR "type ii diabet*" OR "type 2 diabet*" OR "diabetic*" OR "t1dm" **)**	**1,335,680**
	**#3**	**TITLE-ABS-KEY (** "heart disease" OR "vascular diseases" OR "stroke" OR "cerebrovascular accident" OR "sudden death" OR "cardiac arrest" OR "cardiovascular disease" OR "coronary artery disease" OR "heart failure" OR "cardiovascular mortality" OR "coronary death" OR "chd" OR "cvd" OR "cardiac death" OR "myocardial infarction" OR "angina" OR "cad" OR cardiac* OR myocardial* OR "aortic disease" OR "congestive heart failure" OR cardio* OR "ischemic heart disease" OR "atherosclerosis" OR "heart muscle disease" OR "deep vein thrombosis" OR "pulmonary embolism" OR "pericardial disease" OR "rheumatic heart disease" OR "mi" OR "cva" OR "heart" OR "re-infarction" OR re-vascularization OR "cardiac mortality" OR "acute coronary syndrome" OR "percutaneous coronary intervention" OR "arrhythmia" OR "atrial fibrillation" OR "pericarditis" OR "arrhythmia" **)**	**4,564,712**
	**#4**	**TITLE-ABS-KEY (** review OR rat OR rats OR animals OR animal OR in-vitro OR rabbit OR rabbits **)**	**14,828,46**

	#5	**1 AND 2 AND 3 NOT 4**	**4,523**
	#6	**1 AND 2 AND 3 NOT 4 (Limit to Language – English)**	**4,288**

Table S5. Newcastle-Ottawa Scale for assessing the quality.

STUDY	SELECTION (max 4 points)				COMPARABILITY (max 2 points)	OUTCOME (max 3 points)			SCORE (out of 9)
	Representativeness of the exposed	Selection of the nonexposed	Ascertainment of exposure	Demonstration that outcome of interest was not present at start of study	Comparability the basis of the design or analysis	Assessment of outcome	Was follow-up long enough for outcomes to occur?	Adequacy of the follow-up	
**Adderley et al.,**	1	1	1	1	1	1	1	1	8
Aung et al.,	1	1	1	0	1	1	1	0	6
Koo et al.,	1	1	1	0	2	1	1	1	8
Manin et al.,	1	1	1	1	1	1	0	1	7
Rice et al.,	1	1	1	0	1	1	1	1	7
Strausz et al.,	1	1	1	1	1	1	1	1	8
Tahrani etal.,	1	1	1	0	1	1	0	1	6
Wang et al.,	1	1	1	0	1	1	1	1	7
Labarca et al.	1	1	1	1	1	0	1	1	8
